# The EKiTE network (epidemiology in kidney transplantation - a European validated database): an initiative epidemiological and translational European collaborative research

**DOI:** 10.1186/s12882-019-1522-8

**Published:** 2019-10-11

**Authors:** M. Lorent, Y. Foucher, K. Kerleau, S. Brouard, C. Baayen, S. Lebouter, M. Naesens, O. Bestard Matamoros, A. Åsberg, M. Giral, the EKiTE consortium, Anna V. Reisæter, Pål-Dag Line, Anders Åsberg, Dirk Kuypers, Pieter Evenepoel, Kathleen Claes, Bert Bammens, Ben Sprangers, Maarten Naesens, Björn Meijers, Katrien De Vusser, Amaryllis Van Craenenbroeck, Evelyne Lerut, Oriol Bestard Matamoros, Nuria Montero, Salvador Gil-Vernet, Edoardo Melilli

**Affiliations:** 1 CRTI UMR 1064, Inserm, Université de Nantes; ITUN, CHU Nantes; RTRS Centaure, Nantes, France; 2 INSERM UMR 1246 – SPHERE, Nantes University, Tours University, Nantes, France; 3 Centre Hospitalier Universitaire de Nantes, Nantes, France; 4 LabCom RISCA (Recherche en Informatique et en Statistique pour l’Analyse de Cohortes), Nantes, France; 5 Centre d’Investigation Clinique (CIC) en Biotherapie, CHU Nantes, Nantes, France; 6 Department of Microbiology, Immunology and Transplantation, KU Leuven, Department of Nephrology, University Hospitals Leuven, Leuven, Belgium; 7 Nephrology department, Bellvitge University Hospital, Barcelona, Spain; 8 Department of Transplantation Medicine, Oslo University Hospital; Department of Pharmacy, University of Oslo, Oslo, Norway; 9 Inserm Center of Clinic Investigation and Biotherapy, Hôtel Dieu University Hospital, 30, bd Jean Monnet, 44093 Nantes Cedex 01, France

**Keywords:** European database, Kidney transplantation, Cohort, Epidemiology

## Abstract

**Background:**

Kidney transplantation is considered to be the treatment of choice for people with end-stage renal disease (ESRD). However, due to the shortage of available organs and the increase in the ESRD prevalence in Europe, it is essential to improve transplantation outcomes by studying the related prognostic factors. Today, there is no European registry collecting data to perform such clinical epidemiology studies.

**Main body:**

Entitled EKiTE, for European cohort for Kidney Transplantation Epidemiology, this prospective and multicentric cohort includes patients from Spanish (Barcelona), Belgian (Leuven), Norwegian (Oslo) and French (Paris Necker, Lyon, Nantes, Nancy, Montpellier, Nice and Paris Saint Louis) transplantation centers and currently contains 13,394 adult recipients of kidney (only) transplantation from 2005 and updated annually. A large set of parameters collected from transplantation until graft failure or death with numbers of post-transplantation outcomes. The long-term follow-up and the collected data enable a wide range of possible survival and longitudinal analyses.

**Conclusion:**

EKiTE is a multicentric cohort aiming to better assess the natural history of the ESRD in European kidney transplant recipients and perform benchmarking of clinical practices. The data are available for clinical epidemiology studies and open for external investigators upon request to the scientific council. Short-term perspectives are to extend EKITE network to other European countries and collect additional parameters in respect of the common thesaurus.

**Electronic supplementary material:**

The online version of this article (10.1186/s12882-019-1522-8) contains supplementary material, which is available to authorized users.

## Background

In 2016, the European prevalence of End Stage Renal Disease (ESRD) was assessed at 1160 patients per million inhabitants [1]. In many countries, this number is constantly increasing due to the aging population and the increasing prevalence of cardiovascular and metabolic diseases. Fortunately, in several developed countries, including Western Europe, the incidence of ESRD recently stabilized. Nevertheless, the life expectancy of ESRD patients has even increased, resulting in an increase in the ESRD prevalence, a challenge in terms of costs and capacity for health care systems in Europe [2].

Kidney transplantation is considered as the treatment of choice for people with ESRD, because of increased quality of life [3, 4], better chances of survival [5, 6] and lower costs compared to long term dialysis [7, 8]. However, the number of available donor kidneys is not sufficient to meet the demand [9]. One solution is to improve graft survival and to avoid or minimize returns to dialysis and hence better utilize the available organs. For many years, one of the major achievements of clinical and scientific transplantation science has been to decipher the causes of graft rejection and loss and significant improvements have been made. Current ongoing efforts are particularly focused on improving organ failure risk prediction and on identifying the more efficient treatments in real-life settings. Reaching these objectives requires contemporary and high-quality datasets for large cohorts of kidney transplant recipients.

Whilst there are already International and National transplantation registries, such as the Heidelberg-based Collaborative Transplant Study [1], the US Scientific Registry of Transplant Recipients [3, 4] and the ANZDATA registry in Australia and New Zealand [5, 6], no European renal transplantation database exists to enable more precise epidemiology studies, such as Coemans et al. [10]. A European-focused clinical epidemiology study could also help to identify and understand differences in patient care between countries, as recently stated by the European Kidney Health Alliance (EKHA) in their call to improve kidney care in Europe [8].

In this context, we propose a novel cohort entitled “EKiTE” for European cohort for Kidney Transplantation Epidemiology for facilitating European epidemiological studies. It aims to i) provide a better understanding of the evolution of kidney transplant patients, ii) support European guidelines for management of kidney transplant patients (ERBP, European Renal Best Practice), iii) provide data for development or validation of predictive tools, and iv) to support epidemiology studies on rare patient’s profiles or infrequent complications.

In this manuscript, we present the EKiTE cohort by detailing its design and the process for any external researcher requesting access to the data. We also describe its demographic characteristics and provide examples of how the collected data can be used to foster new research opportunities.

## Construction and content

### Inclusion criteria

The EKiTE cohort was set up to combine into a single European cohort all incident adult kidney (only) transplant recipients since 2005 and updated annually. Only patients with negative crossmatches at the time of surgery were included. To date, the data are collected from existing databases from 4 countries: 1) the Spanish monocentric cohort of Bellvitge University Hospital Transplant Registry in Barcelona (*n* = 840), 2) the Belgium monocentric cohort of Biobank Renal Transplantation of the University Hospitals Leuven (*n* = 1684), 3) the French multicentric DIVAT cohort (Données Informatisées et Validées en Transplantation, *n* = 8433) based on 7 transplantation centers (Paris Necker, Lyon, Nantes, Nancy, Montpellier, Nice, Paris Saint Louis), and 4) the Norwegian Renal Registry of the University Hospital of Oslo (which performs all Norwegian kidney transplantations, *n* = 2437).

### Follow-up

Patients are followed until death or graft failure (i.e. definitive return to dialysis or pre-emptive re-transplantation). For patients alive at the time of the data importation, the maximum follow-up time corresponds to the time between the surgery and the last date for which we know that the recipient is alive with a functioning graft. All patients are informed of the data collection and give informed consent, if required by the national or local ethical committee.

### Data collection

As mentioned above, individual patient data entered into the EKiTE database has already been collected in the participating centers and therefore data collecting procedures currently performed according to local practices are unchanged. We voluntarily limited data collection to 44 informative parameters related to kidney recipients and donors that we considered essential to perform epidemiologic analyses. The data are listed in Table 1 along with their common definition in the EKiTE thesaurus.

**Table 1:** List of variables included in EKiTE

	Variable	Definition
Variables related to the recipient of the surgery	Year of transplantation	Calendar year of the current transplantation
	Recipient gender	Gender of the recipient
	Recipient age	Recipient age in years at transplantation
	Recipient height	Recipient height in centimeters at transplantation
	Recipient weight	Recipient weight in kilograms at transplantation
	Duration under renal replacement therapy	Number of days between the first dialysis (hemodialysis and/or peritoneal dialysis) or the first preemptive transplantation and the transplantation
	Duration on waiting list before the transplantation	Number of days between the registration on the waiting list related to the current transplantation and the transplantation
	Type of dialysis just before the transplantation	Type of extra renal purification technique just before the transplantation
	Primary renal disease	Primary cause of renal failure
	Vascular history	Whether the recipient had a history of vascular disease before transplantation
	Cardiac history	Whether the recipient had a cardiovascular history before transplantation
	Cancer history	Whether the recipient had cancer before transplantation
	Diabetes history	Whether the recipient had diabetes before transplantation
	Hepatitis history	Whether the recipient had hepatitis B or C before transplantation
	Recipient CMV serology	Result of the last recipient cytomegalovirus serology before transplantation
	Recipient EBV serology	Result of the last recipient Epstein-Barr virus serology before transplantation
	Anti-class I Immunization	Whether anti-HLA (Human Leukocyte Antigen) class I antibodies were detected during the 6 months before transplantation
	Anti-class II Immunization	Whether anti-HLA (Human Leukocyte Antigen) class II antibodies were detected during the 6 months before transplantation
	Recipient HIV serology	Result of the last recipient human immunodeficiency virus serology before transplantation
	Recipient blood group	Blood group of the recipient
	Induction therapy	Type of induction therapy at transplantation
Variables related to donor at surgery	Age at the graft retrieval	Donor age in years at kidney retrieval
	Donor gender	Gender of the donor
	Type of donor	Whether the transplant came from a living or deceased donor
	Type of deceased donation	Heart-beating status for the deceased donations
	Donor cause of death	Indication of the cerebrovascular cause of death of the donor
	Last donor serum creatinine	Last serum creatinine level in μmol. L^− 1^ of the donor before graft retrieval
	Donor CMV serology	Result of the last cytomegalovirus serology of the donor before graft retrieval
	Donor EBV serology	Result of the last Epstein-Barr virus serology of the donor before graft retrieval
	Donor blood group	Blood group of the donor
Variables related to transplantation at surgery	Cold ischemia time	Time in minutes between the graft retrieval and its reperfusion, the warm ischemia time being excluded
	Number of HLA-A mismatches	Number of mismatches between the donor and the recipient concerning Human Leukocyte Antigen Locus A (HLA-A)
	Number of HLA-B mismatches	Number of mismatches between the donor and the recipient concerning Human Leukocyte Antigen Locus B (HLA-B)
	Number of HLA-DR mismatches	Number of mismatches between the donor and the recipient concerning Human Leukocyte Antigen Locus DR (HLA-DR)
	Rank of transplantation	Rank of the current kidney transplantation, i.e. number of previous kidney transplantations + 1
Variables collected during the post-transplantation period	Recipient weight	Recipient weight in kilograms at 3 months and 6 months post-transplantation, and at each anniversary of the transplantation
	Maintenance immunosuppressive drug (7 subtypes)	Maintenance immunosuppressive drug prescribed at 3 months and 6 months post-transplantation, and at each anniversary of the transplantation. In case of switching, the most recent treatment is indicated. 7 treatments may be indicated: calcineurin inhibitor, mycophenolate mofetil, mycophenolic acid, azathioprine, sirolimus, everolimus and steroids
	Recipient serum creatinine	Recipient serum creatinine level in μmol. L^−1^ at 3 months and 6 months post-transplantation, and at each anniversary of the transplantation
	Recipient daily proteinuria	Recipient proteinuria in g.L^−1^ at 3 months and 6 months post-transplantation, and at each anniversary of the transplantation
	Delayed graft function	The need for at least one dialysis session within the first 7 days post-transplantation
	Time-to-death	Time in days between the surgery and the patient’s death with a functioning graft (death after return-to-dialysis is not considered)
	Time-to-graft failure	Time in days between the surgery and the definitive return to dialysis or pre-emptive transplantation
	Time-to-first acute rejection episode	Time in days between the surgery and the first acute rejection episode
	Maximum follow-up time	Time in days between the surgery and the last known date at which the recipient was alive with a functioning graft

### Data circuit and production software

**Fig. 1:**
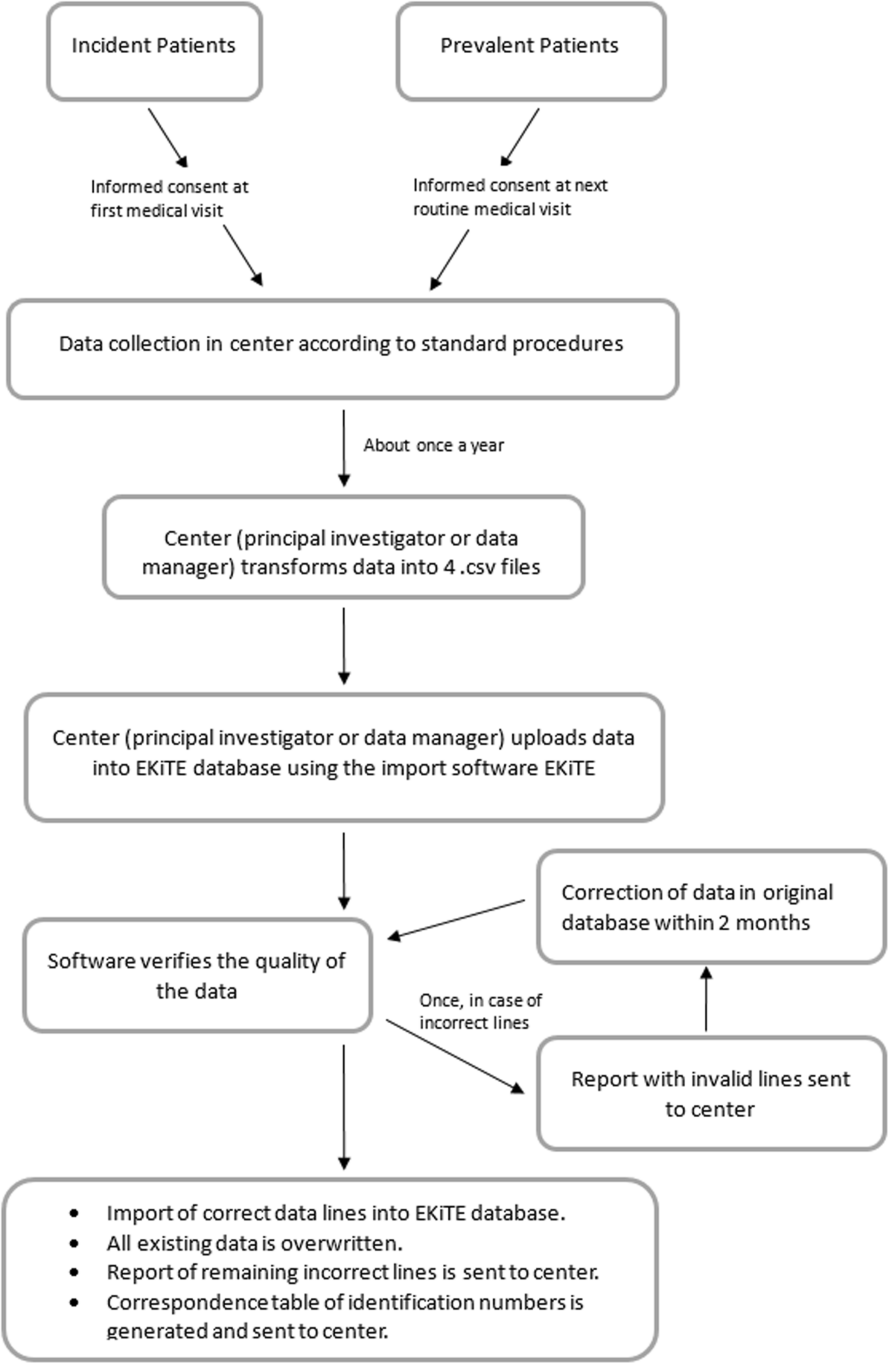
Flowchart of data collection process

The principal investigator or the data manager from each local cohort extracts annually the required parameters from their own local database using a standard *.csv* electronic format and uploads it into the EKiTE data warehouse using an original software program specifically developed by the IDBC Company (www.idbc.fr). The principle is to automatically convert the parameters into a common coding system according to the EKiTE thesaurus (Table 1) and control the correctness of the uploaded data. As illustrated in Fig. 1, a report of incorrect data is produced when databases are uploaded, such that corrections can be made in the original cohort. This process allows to considerably limiting outliers and incoherent data. Data are stored in the EKiTE data warehouse using an Oracle platform designed to meet the highest security standards and are accessible via a secure Windows 2008 Web Server hosted by the company IDBC/A2COM (with the French label for data hosting, *Hébergeur de Santé*).

### Data coding

For each patient a unique identification key is generated for the EKiTE database. Only the principal investigator from the center in which the patient was transplanted can make the correspondence between the EKiTE key and to the original cohort key. To ensure that patients cannot be identified in any way, dates are converted to a corresponding time post-transplantation. Overall, the included data exclude any information that might directly or indirectly identify a patient.

### Quality control

The quality of data is verified respecting each cohort local procedures. Missing data is closely monitored, and it is strongly encouraged that the data are retrieved if possible using a list of base variables that is pre-specified by the Scientific Committee. Inspection or audit by health authorities may take place. The coordinator and/or participating centers must be able to provide data access to inspectors or auditors.

### Regulatory aspects

A consortium agreement has been written and approved by the Directors of each University Hospital participating in the EKiTE network. It stipulates the specific and mandatory rules for a common networking. Collected data are stored in a computer system respecting the French law on Information technology and civil liberties (January 6th 1978, amended in April 29th 2004). The protocol received authorization from the CCTIRS (*Comité Consultatif sur le Traitement de l’Information en matière de Recherche dans le domaine de la Santé*) as well as the CNIL in December 2017 (*Commission Nationale de l’Informatique et des Libertés*, N°917155).

### Data extraction procedure and software

Each center has the right to login to the database to access their own data (using a personal username and password). Data from other centers is not accessible. The network manager is the only person with access to all data using a personal username and password.

The Plug-Stat® software developed by the common laboratory in Research in Informatics and Statistics for Cohort-based Analyses (LabCom RISCA, www.labcom-risca.com/plug-stat) is an internet-accessible application running on the main web browsers. It allows to select a sample of patients according to inclusion criteria and then to perform various queries such as counting the number of patients, extracting the data, comparing the distribution of a variable between two exposure groups (i.e. two sub-groups of patients among the patients respecting the inclusion criteria), computing and plotting various statistical indicators (means, proportions and survival curves). Beside these usual descriptive methods, Plug-stat® also allows taking into consideration the main difficulties of long-term observational studies: confounding factors, competing events and longitudinal markers. The results are directly exploitable for publication in international peer review journals.

Via Plug-Stat®, each center has the right to login to the database to access their own data (using a personal username and password). Data from the other centers is not accessible, except for a particular authorization by the network manager and the scientific council for a particular multicentric study. The network manager is the only person with a permanent access to all data and is in charge of setting up the different authorization for extracting the data and for authorizing the mono or multi-centric accesses.

### Governance and working rules of the network

#### Governance

The network is regulated by the following personnel: one scientific coordinator, one database administrator, four scientific managers (one per country), and one methodologist/biostatistician. The Steering committee is composed of the coordinator and the four managers. The steering committee members meet once per year to plan the Network’s activities, to implement the database and to validate the quality procedures. The scientific committee is composed of the coordinator, the four scientific managers and four statisticians/epidemiologists (one per country). Scientific Committee members meet once per year to approve scientific projects and discuss various strategic decisions.

#### Publication rules

Any publications that make use of the EKiTE data from one or several centers must cite the center initiating the study, and all the centers involved in the study. Author positions in the publication are attributed according to the scientific involvement of each center. All co-authors are supposed to read and approve the initial study protocol and the final version of the manuscript before publication.

Any publication/communication must mention the EKiTE Network and relevant financial support (institutional or private). Finally, any publication/communication must mention in the acknowledgments all the research staff involved in the network (data managers, statisticians, clinical research associates, etc.).

#### Open data rules

**Fig. 2:**
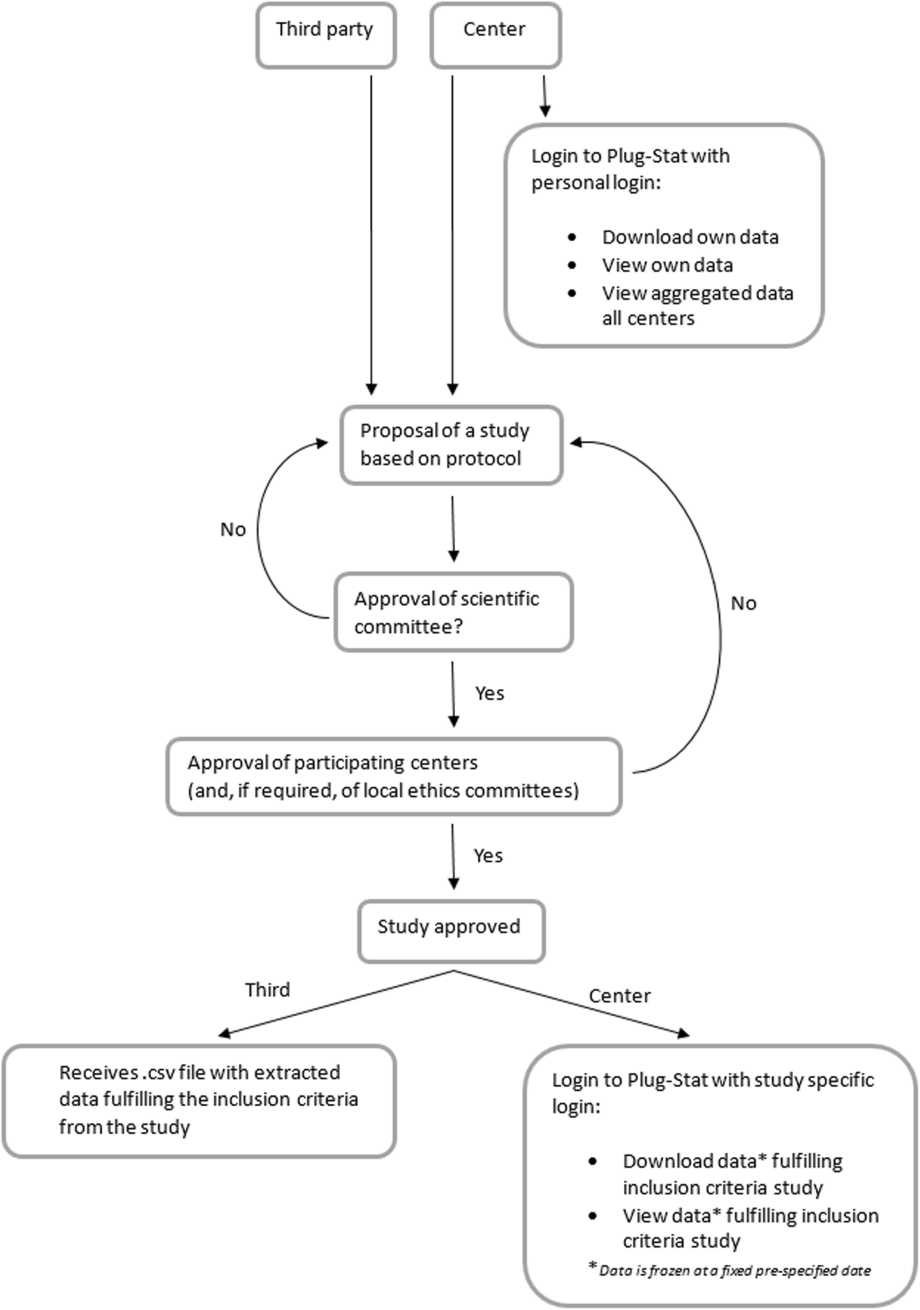
Flowchart of data access procedure

Each participating investigator has the right to login (using a personal username and password) to the data of his own center. A participating or a third (meaning external to the EKiTE network) investigator may request access to the database to perform a scientific project. In this case, a project proposal is sent by mail (magali.giral@chu-nantes.fr) to the Scientific Committee of the network. If the project is approved by the Scientific Committee, and thereafter by the relevant local ethics committees of centers whose data is used, the study is definitively approved and the study can start, as illustrated in Fig. 2. In the case of non-approval of the project by the Scientific Committee or one of the ethics committees, a modified study proposal can be submitted.

In case of approval for a participating investigator, she/he receives a study ID and password with which she/he can login to the database via the software Plug-Stat®. This gives access to the data from the other centers. The data can be analyzed directly using Plug-Stat® or can be downloaded to perform analysis using an external statistical software. For a third investigator, she/he will receive an electronic extraction of the database in .csv format, including the data from the requested centers that fulfill the requested inclusion and exclusion criteria.

The network manager will be the sole person which has access to all data using a personal username and password. In any case, data are only shared in a way such that their confidentiality are preserved.

## Utility and discussion

### Description of the cohort at baseline

At present a total of 13,394 kidney transplantations between 2005 and 2018 are included in EKiTE. The demographics and baseline characteristics of recipients and donors at the time of transplantation are presented in Table 2. The mean recipient age was 52.3 (± 14.0) years and 63.5% were male. History of vascular disease, cardiac disease, diabetes and cancer concerned respectively 14.9, 24.6, 17.4, and 11.9% of the recipients before transplantation. 17.6% of the transplants were repeat transplantation after failure of a previous graft. 16.2% of the transplantations were pre-emptive, prior to initiation of dialysis. Immunologic characteristics included: 23.9, 39.7 and 13.8% of patients presenting more than 1 HLA–A, −B or -DR incompatibility respectively, 36.0% were immunized against class I HLA antigens prior to transplantation and 32.5% against class II. The mean cold ischemia time was 11.0 (± 10.3) hours, and glomerulonephritis was diagnosed as initial nephropathy in 27.9% of the recipients. Donors were mainly deceased (81.5%) with a mean age of 52.1 (± 15.8) years and 54.8% were male. The mean last donor serum creatinine was 84.8 (± 50.3) μmol. L^− 1^ and cause of death was cerebrovascular damage in 46.5%.

**Table 2:** Description of the EKiTE cohort (*N* = 13,394)

Continuous variables: mean ± sd	Missing data		Total cohort	
Categorical variables: number (%)	*N*	(%)	(*N* = 13,394)	
TRANSPLANTATION				
Year of transplantation > 2010	–	–	7894	(58.9%)
Rank of the graft ≥2	–	–	2353	(17.6%)
Cold ischemia time (min)	151	(1.1)	661.3	±618.6
HLA-A mismatches > 1	549	(4.1)	3075	(23.9%)
HLA-B mismatches > 1	314	(2.3)	5188	(39.7%)
HLA-DR mismatches > 1	402	(3.0)	1799	(13.8%)
RECIPIENT				
Male gender	–	–	8500	(63.5%)
Age (years)	–	–	52.3	±14.0
Height (centimeters)	935	(7.0)	169.6	±9.8
Weight (kilograms)	1290	(9.6)	72.0	±15.4
Dialysis (years)	368	(2.7)	3.4	±4.6
Duration on waiting list (years)	3649	(27.2)	2.0	±2.0
Type of dialysis				
No dialysis	29	(0.2)	2159	(16.2%)
Hemodialysis			9580	(71.7%)
Peritoneal dialysis			1626	(12.2%)
Primary renal disease				
Glomerulonephritis	1910	(14.3)	3200	(27.9%)
Tubulo interstitial disease			4035	(35.1%)
Reno-vascular disease			1054	(9.2%)
Diabetes			1075	(9.4%)
Other disease and unknown			2120	(18.5%)
Vascular history	33	(0.2)	1992	(14.9%)
Cardiac history	96	(0.7)	3267	(24.6%)
Diabetes history	–	–	2325	(17.4%)
Cancer history	5	(0.0)	1595	(11.9%)
Positive CMV serology	332	(2.5)	8722	(66.8%)
Positive EBV serology	5041	(37.6)	8096	(96.9%)
Positive HIV serology	1801	(13.4)	129	(1.1%)
Positive anti-class I HLA antibodies	2838	(21.2)	3797	(36.0%)
Positive anti-class II HLA antibodies	2967	(22.2)	3388	(32.5%)
Blood group				
O	7	(0.0)	5478	(40.9%)
A			5889	(44.0%)
B			1431	(10.7%)
AB			589	(4.4%)
Induction therapy				
No induction	65	(0.5)	802	(6.0%)
ATG			4987	(37.4%)
Anti-IL2R			6680	(50.1%)
Belatacept			7	(0.0%)
Other			853	(6.4%)
Delayed Graft Function	1727	(12.9)	2703	(23.2%)
DONOR				
Age at retrieval (years)	148	(1.1)	52.1	±15.8
Male gender	111	(0.8)	7283	(54.8%)
Type of donor				
Deceased	74	(0.6)	10,855	(81.5%)
Living			2465	(18.5%)
Type of deceased donor				
Heart beating	12	(0.1)	10,278	(76.8%)
Non-heart-beating			635	(4.7%)
Non-attributable			2469	(18.5%)
Donor cause of death				
Cerebrovascular	171	(1.3)	6146	(46.5%)
Other			4608	(34.8%)
Non-attributable			2469	(18.7%)
Last donor serum creatinine (μmol/L)	2119	(15.8)	84.8	±50.3
Positive CMV serology	135	(1.0)	7720	(58.2)
Positive EBV serology	4428	(33.1)	8518	(95.0%)
Blood group				
O	56	(0.4)	6032	(45.2%)
A			5670	(42.5%)
B			1192	(8.9%)
AB			444	(3.3%)

### Description of the outcomes

The median follow-up time was 4.1 years (range from 0.0 to 13.3). Delayed Graft Function (DGF) occurred in 2703 patients (23.2%). The mean recipient serum creatinine was 139.2 (± 56.8) μmol. L^− 1^ at 3 months post-transplantation, 139.1 (± 55.6) μmol. L^− 1^ at 6 months and 138.3 (± 55.8) μmol. L^− 1^ at 1 year. Approximately 6.3% had a 1-year proteinuria higher than 1 g/24 h. Finally, 16.2% of the recipients presented at least one episode of acute rejection (ARE) before the first anniversary of the graft.

**Fig. 3:**
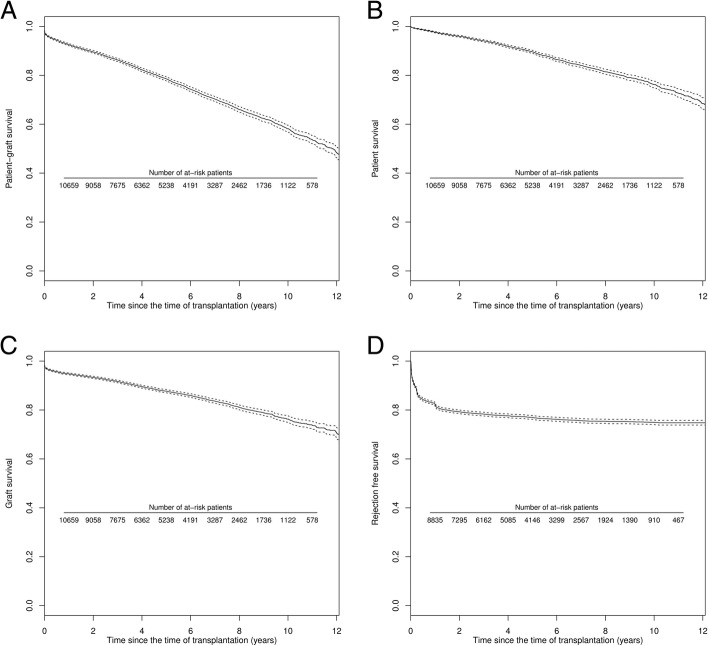
Survival curves according to the time of transplantation (*n* = 13,394) from Kaplan-Meier estimator (black lines) and their corresponding 95% CI (dotted lines). **a** Patient-graft survival; **b** Patient survival (with a functioning graft); **c** Graft survival (with death-censored); **d** Rejection free survival

Several survival curves and their corresponding 95% confidence interval (95% CI) are presented in Fig. 3 (Kaplan-Meier estimator). At 10 years post-transplantation, 1637 recipients returned to dialysis, 1396 died with a functioning graft and 1122 remained alive. Patient-graft survival at 10 years after the transplantation was 58.1% [95% CI 56.7%; 59.6%]. The corresponding patient and death censored graft survival were 76.2% [95% CI 74.9%; 77.6%] and 76.3% [95% CI 75.0%; 77.6%] respectively. Finally, acute rejection episode-free survival at 2-years post-transplantation was 79.0% [95% CI 78.5%; 79.6%].

### Examples of future scientific projects

A project that will be performed soon is the external validation of several prognostic scores in kidney transplantation, notably those developed by the DIVAT-SPHERE group in Nantes:

- The Kidney Transplant Failure Score (KTFS) [11] is a composite score, taking into account a series of eight well-accepted pre- and post-transplant risk factors of graft loss, which evaluates the risk of graft failure up to 8 years post transplantation. The clinical utility of the KTFS is currently under study in a randomized clinical trial (national PHRC 2011 - TELEGRAFT, clinicaltrials.gov: NCT01615900) [12]. The objective is to demonstrate the efficiency of personalized care according to the KTFS.
- The Delayed Graft Function Score (DGFS) [13] is a simple composite score to classify patients according to their DGF (Delayed Graft Function) risk at the time of transplantation and to thus propose individualized management or therapeutic strategies. The clinical utility of the DGFS is currently under study in a randomized clinical trial (national PHRC 2013 – PREDICT-DGF, clinicaltrials.gov: NCT02056938) [14]. The aim is to evaluate the effectiveness of ATG (Anti Thymocyte Globulins) for preventing DGF in patients with low immunologic risk and high risk of DGF, in comparison with Simulect treatment.
- The 1-year Recipient Risk Score (RRS) [15] includes five pre- and post-transplant variables and is intended to predict mortality after the first year of transplantation. The integration of the 1-year RRS in the TELEGRAFT clinical trial could be complementary to the KTFS. This would allow adapting patient care according to the risk of death and the risk of graft loss.

These scores seem to be robust, since; i) they were developed on the basis of high-quality data, ii) they are based on adapted statistical modeling, and iii) they are validated on independent data. The external validation (European patients) of these scores is essential to evaluate their applicability outside of France and for an international recognition of these studies.

An important objective of EKiTE is to allow access to the data warehouse to support projects by internal or external teams. The following studies could be performed: the comparison of baseline characteristics of transplanted patients between the 4 countries, assessment of the disparity in treatments and prescriptions between the 4 countries, the study of the determinants of the net survival, i.e. the survival when the only possible deaths are related to ESRD [16], and the role of the organ donor’s last serum creatinine level prior to retrieval in the prognosis of recipient and graft survivals.

### Limits of the collected data

For the first step in the construction of the EKiTE cohort, we have chosen to limit the number of collected data that are not subject to discussion or clinical interpretation. It is made mainly with general characteristics of the transplantation, the donor and the recipient at the time of the surgery and some of them during the post-transplantation period. Additional data such as type of rejection (cellular or antibody mediated), biopsy findings or de novo anti HLA immunization (in particular donor specific) will be useful for future epidemiological studies. A perspective of the network will be to work progressively to increase the number and the precision of the collected parameters. However, since several centers that participate to the EKITE cohort [17] do not practice surveillance biopsies it makes difficult to enter pathology findings on the database. Concerning DSA immunization, it was not possible to retrospectively collect this parameter. Nevertheless, because DSA are now routinely diagnosed at the time of transplantation and at each anniversary of the transplantation in all participating centers of the EKITE cohort, it would be possible in a next step to collect this parameter for incident kidney transplant recipients from 2020, with also anti HLA specificity and MFI.

## Conclusion

EKiTE is a novel, large European multinational cohort with a large amount of baseline data from the recent era of kidney transplantation that is updated annually and has a low level of missing data. It will allow epidemiologic studies to be performed to better assess the western European transplantation patient profile and allow benchmarking to improve clinical practices. Other European centers are invited to join the EKiTE network. For details on the process of inclusion of a new center you can take note of the “Consortium Agreement for the EKiTE network” (provided in Additional file 1) or make contact with a network member at the following address www.labcom-risca.com/ekite-en*.* External investigators of the EKiTE network, are also welcome to ask for access to this open database for scientific works.

## Additional file

Additional file 1: Consortium Agreement for the EKiTE network. (DOCX 501 kb)

## Data Availability

The EKITE database analyzed during the current study were obtained from 4 existing datasets: the Spanish monocentric cohort of Bellvitge University Hospital Transplant Registry in Barcelona, 2) the Belgium monocentric cohort of Biobank Renal Transplantation of the University Hospitals Leuven, 3) the French multicenter DIVAT cohort (www.divat.fr) and 4) the Norwegian Renal Registry of the University Hospital of Oslo. There is public access to the database available from the corresponding author on reasonable request and restricted to the approval of the scientific committee of the EKITE consortium and thereafter by the relevant local ethics committees of centers whose data is used. The EKITE database received administrative permission to access and to use these datasets.

## Abbreviations

ANZDATA: Australia and New Zealand Dialysis and Transplant Registry
ARE: Acute Rejection Episode
CCTIRS: Comité Consultatif sur le Traitement de l’Information en matière de Recherche dans le domaine de la Santé
CI: Confidence Interval
CMV: Cytomegalovirus
CNIL: Commission Nationale Informatique et Libertés
DGF: Delayed Graft Function
DIVAT: Données Informatisées et VAlidées en Transplantation
DSA: Donor Specific Antibodies
EBV: Epstein Barr Virus
EKHA: European Kidney Health Alliance
EKiTE: Epidemiology in Kidney Transplantation - a European validated database
ERBP: European Renal Best Practice
ESRD: End Stage Renal Disease
HIV: Human Immunodeficiency Virus
HLA: Human Leukocyte Antigen
MFI: Mean Fluorescence Intensity
RISCA: Research in Informatics and Statistics for Cohort-based Analyses
sd: Standard Deviation
