# Supplementary material for: The EKiTE network (epidemiology in kidney transplantation - a European validated database): an initiative epidemiological and translational European collaborative research
Source: BMC Nephrol. 2019 Oct 11;20:365. doi: 10.1186/s12882-019-1522-8 (PMC6788117; doi:10.1186/s12882-019-1522-8)
Supplement: Supplementary file 1 — Consortium Agreement for the EKiTE network. (DOCX 501 kb) [file 12882_2019_1522_MOESM1_ESM.docx]

**Consortium Agreement**

**for the EKiTE network**

**Coordinator: Magali Giral**

# Between

**Nantes University Hospital**

A public health establishment

Whose registered office is at 5 allée de l’Île Gloriette – 44093 Nantes Cedex 1 - France Represented by its Chief Executive Officer, Mr PHILIPPE SUDREAU

Hereinafter "**the UH of Nantes** or **Centre 1”** "

Acting on behalf of the Centre of Research in Transplantation and Immunology (ITUN) and in the presence of Ms. Magali Giral, hereinafter referred to as "the Coordinator".

And

# Oslo University Hospital

A public health establishment

Whose registered office is at P. O. Box 4950 Nydalen - N-0424 Oslo - Norway Represented by its Chief Executive Officer, Mr BJØRN ERIKSTEIN

Hereinafter "**the UH of Oslo** or **Centre** 2”

Acting on behalf of the Transplantation Medicine department and in the presence of Mr. Anders Åsberg And

# University Hospital Leuven

A public health establishment

Whose registered office is at Herestraat 49 - 3000 Leuven - Belgium Represented by its Chief Executive Officer, Mr MARC DECRAMER Hereinafter "**the UH of Leuven** or **Centre** 3”

Acting on behalf of the Nephrology department and in the presence of Mr. Maarten Naesens And

# Bellvitge University Hospital

A public health establishment

Whose registered office is at Feixa Llarga - s/n L'Hospitalet de Llobregat – 08907 Barcelona - Spain Represented by its Chief Executive Officer, Mr ANTONI LLUÍS ANDREU PÉRIZ

Hereinafter "**the UH of Barcelona** or **Centre** 4”

Acting on behalf of the Nephrology department and in the presence of Mr. Oriol Bestard

Hereinafter referred to individually as "the **Party** or the **Centre**" or collectively "the **Parties** or the

**Centres**".

Initials CHU Nantes Page 1 / 19 initials Parties Created by CJ CHUN

**Considering**

- Country specific legislation on health and patients’ rights The Public Health Code;
- EU data protection legislation (European Directive 95/46-CE) as well as its implementing national laws;
- Country specific legislation on clinical research
- EU legislation regarding intellectual property as well its implementing national laws.

# As a whole, legislation as well as its implementing texts of each participating country.

**PREAMBULE:**

**Scientific summary of the project:**

Kidney transplantation is considered the treatment of choice for people with end-stage kidney failure, because quality of life and chances of survival are often favorable compared to people who are treated with dialysis. However, in practice the possibility to transplant kidneys is limited, due to a shortage of available organs. It is therefore essential to make kidney transplantations as efficient as possible. To do so, two main objectives can be identified: 1) to identify the factors that influence whether transplantation will be successful or not, and 2) to adapt the care of organ recipients based on the risk of particular complications. The first objective will enable an allocation of donated organs to those who will benefit most and the second objective will prolong the survival of transplanted kidneys and delay potential re-transplantations.

To fulfil these objectives, several methods have already been proposed. However, these methods have typically been developed and validated on national data. Since patient populations, culture and treatment regimens differ per country; such methodology can typically not reliably be used in other countries without validating it on local data. Also, national data bases do not always contain enough data to evaluate the impact of rare events on the success of kidney transplantation. Therefore the objective of the EKiTE (Epidemiology in Kidney Transplantation - a European validated database) network is to set up a European database of kidney transplant patients with high data quality. This European database will aid to

- provide an international validity to existing clinical research methods for kidney transplant patients.
- perform epidemiological studies on rare patient profiles or infrequent complications due to the large cohort size.
- better understand the evolution of kidney transplant patients in Europe and compare this to the information that is available from the North-American and Australian registries.
- support the European guidelines for management of kidney transplant patients (ERBP, European Renal Best Practice).

The Centers wish to establish a collaboration between each other in the field of renal transplantation, in order to:

- - organize relations between the Centres;
  - collect and exchange information;
  - initiate and conduct studies e.g. jointly;
  - use the results for publications intended for the scientific community;
  - use the results for potential partnerships with the pharmaceutical industry.

The Parties have decided to create a cohort of patients through the use of a centralized database within the context of their network (hereinafter referred to in this agreement as "EKiTE Network" or “EKiTE” or “the Network”) which is organized as follows:

- - - a central database, accessible online, is produced in Nantes;

Initials CHU Nantes Page 2 / 19 initials Parties Created by CJ CHUN

- - - the data collected in this centralized database are collected and uploaded to the central database by the local staff of each Centre.

The purpose of the EKiTE Network is to enable:

- - - - the data to be secured;
      - single maintenance and update;
      - high quality data;
      - the advancement of clinical and methodological research based on the data in the centralized database.

The EKiTE Network has a fixed starting budget presented in Annex 1. The EKiTE Network has specific funding.

**Article – 1. Purpose**

The purpose of this agreement (hereinafter referred to as the "Agreement" or the "Consortium") is to organize relations between the Parties within the context of the Project, and, in particular to:

- - lay down their rights and obligations,
  - organize the governance of the Project,
  - establish the rules of ownership and exploitation of the results of the Project.

**Article - 2.1. Composition**

EKiTE is composed, on the day of the signing of this Agreement:

- - - of Nantes University Hospital; *ITUN*;
    - of Oslo University Hospital*; Department of Transplantation Medicine;*
    - of University Hospital Leuven; *Nephrology department;*
    - of Bellvitge University Hospital; *Nephrology department.*

Other Centers may join the Network in accordance with the conditions of Article 2.4 of this Agreement.

**Article – 2.2. Scientific Managers**

Each Centre will designate one or more Scientific Managers. On the day this Agreement is signed, the Scientific Managers appointed by the Centres are:

- - - - For Nantes: Ms. Magali Giral;
      - For Oslo: Mr. Anders Åsberg;
      - For Leuven: Mr. Maarten Naesens;
      - For Barcelona: Mr. Oriol Bestard.

If a Centre's Scientific Manager is replaced during the term of the Agreement, the Centre in question will inform the Coordinator in writing (email or letter) as soon as possible so that she can inform all the Centers.

**Article – 2.3. Organisation of the Network**

## STEERING COMMITTEE:

*Composition*

- - The Coordinator;
  - The Centers’ Scientific Managers, or their representatives as mentioned in Article 2.2;
  - Invited members: clinical research assistant, data managers, and Centers’ physicians.

Initials CHU Nantes Page 3 / 19 initials Parties Created by CJ CHUN

*Mission statement*

- Planning of the Network's activities;
- Organization;
- Implementation of the quality procedure;
- Implementation of the Database.

*Meetings:* 1 time per year.

The Steering Committee meets validly when at least half of the members are present or represented. Within the Steering Committee, decisions are taken by a simple majority of the votes of the members present or represented. Invited members do not vote. In the event of a tie, the Coordinator has the casting vote. The Coordinator shall provide minutes of each meeting of the Steering Committee and send them by email to each Centre's Scientific Manager for information. These documents will be archived in the Nantes Centre which will be responsible for acting as the Network's secretariat and for keeping its archives.

## SCIENTIFIC COMMITTEE:

*Composition*

- The Coordinator
- The Scientific Managers of each Centre, or their representative
- Statisticians/epidemiologists of each Centre
- Invited members: Centers’ physicians

*Missions*

- Assessment of applications to perform scientific projects using the data from the EKiTE database;
- Scientific assessment of the projects that make use of the data from the EKiTE database;
- Determination of the rights, the use and the provision of data in the context of studies carried out by the Network's Centers in accordance with Article 6;
- Determination of the conditions of access and provision of data by public or private third parties in accordance with Article 7.3;
- Make arrangements for the inclusion of new Centers and the exclusion of participating Centers;
- Advice regarding the technological and strategic investments;
- Plan the budgetary breakdown of the funding obtained;
- Assessment of the results of the activity;
- Decide how to promote the results of scientific projects that have been performed using the data from the EKiTE database, in accordance with Article 6.

*Meeting:* 1 time per year

The Scientific Committee meets validly when at least half of the members are present or represented. Within the Scientific Committee, decisions are taken by a majority of the votes. Invited members do not vote. In the event of a tie, the Coordinator has the casting vote. The Coordinator shall provide minutes of each meeting of the Scientific Committee and send them by email to each Centre's Scientific Manager for information. These documents will be archived in the Nantes Centre which will be responsible for acting as the Network's secretariat and for keeping its archives.

**Article – 2.4. Inclusion of new Centers in the network**

Other Centers will be able to become part of the Network. In order to become part of the Network, each applicant must submit its written and argued application to the Coordinator who will present it to the Scientific Committee. Acceptance of a new Center into the Network will be subject to the agreement of the Centers already included in the Network, through the Scientific Committee, under the conditions laid down in Article 2.3 and must be formalized by an addendum to this Agreement, signed by all of the Parties.

Initials CHU Nantes Page 4 / 19 initials Parties Created by CJ CHUN

In any event, opening up the Network to third party Centers which so request, will not be considered until after the first quality evaluation of the Network (carried out under the conditions specified in Article 4.2 of this agreement).

**Article – 3. Compliance of the EKiTE Network with the legal and regulatory requirements**

The Nantes Centre, the Coordinator, has undertaken to implement all the regulatory measures necessary to guarantee compliance of the EKiTE database with French law, and therefore to obtain all opinions, declarations, authorizations and decisions required for the EKiTE database to be carried out on its site.

Each participating Centre is responsible for the completion of the legal formalities and regulations relating to data entered into the EKiTE database, as well as the collection of the data within it, the collection of human biological samples and obtaining the opinions, declarations or authorizations relating thereto.

In particular and if required, each Centre is responsible for informing and obtaining the consent of the persons whose personal data are collected within the EKiTE Network and whose biological samples constitute the shared virtual biocollection. Each Centre is responsible for the collection and treatment of the samples regarding its patients, before sending them, as well as informing and obtaining their consent, if required. Each Centre becomes responsible for the samples received from another Centre upon their receipt.

If applicable, the Scientific Managers of the Centers in collaboration with the research staff of each Centre must check the reality of the signed consent for the patients, prior to recording the patients in the database or prior to transferring biological samples. The patient's medical/clinical/biological data will be coded on entry into the database.

Each Centre will have an access code to the EKiTE database.

**Article – 4. Creation of the database**

The Centers undertake to enter data into the EKiTE database according to the procedures described in Article 4.2 of this document.

**Article – 4.1. Procedures for entering data.**

Each Centre's research staff or Scientific Managers of the Centers will enter, once per year, the data listed in Annex 2.

**Article – 4.2. Quality procedures and standards**

All items will be coded, using automatic conversion, in accordance with the Thesaurus developed in agreement with all the Centres. Modifications in the database will be logged with their modification date. Missing data will not be allowed for the set of baseline items specified in Annex 2. For the remaining items, missing data is only allowed if it has been verified that the missing values cannot be obtained. The correctness of the data will be assessed using quality monitoring systems implemented in a software program which manages the EKiTE data. Once per year, an extensive quality evaluation of all data that was entered since the previous quality evaluation will be performed using this software program.

**Article – 5. Constitution of the shared virtual Biocollection**

The EKiTE Network may require the constitution of a virtual biocollection using human biological samples from local collections of the Centers. It is understood that each Centre manages its own biocollection as usual and is responsible for the collection and treatment of the samples regarding its patients**.**

Initials CHU Nantes Page 5 / 19 initials Parties Created by CJ CHUN

Some biological samples may be shared for specific scientific projects, after Scientific Committee approval and after opinions, declarations, authorizations and decisions if required. In this context, a mutual/material transfer agreement will be signed by all Parties before the beginning of the project.

Each Centre is free to use its own biological samples for its own projects.

**Article – 6. Operation of the EKiTE Network**

**Article – 6.1. Protection of the Centers with respect to the rights of data producers**

It is recalled that each Centre is responsible for the quality of its data and must accept to undergo the quality control procedures described in Article 4.2 of this Agreement.

The UH of Nantes is the producer of the Network's centralized database and shall therefore be considered as the data controller as defined in the EU Data Protection Directive 95/46/EC. Within the context of the collaboration of the Centers in EKiTE, each Centre has the power to control the extraction and use of the data from its Centre in accordance with the conditions laid down in the following articles.

**Article – 6.2. General conditions of extraction and use of the Centers’ data.**

In accordance with Article 7.2, each Centre is free to use its own data for its own projects.

A Centre that wishes to extract or to use the data of one or more other Centers must make a written request (email or letter) to the Coordinator. This request includes a description of the scientific project. The said extraction or use can only be performed after written agreement (email or letter) from the Centre or Centers from which the data originate and that of the Scientific Committee.

Each Centre initiating a scientific project based on the use of EKiTE data undertakes to find the necessary funding to complete its project and to implement all the regulatory procedures necessary to ensure the compliance of its project with the legislation/regulations in force, and thus request all opinions, declarations, authorizations and decisions required. Studies made jointly between several Centers must be subject to written methodological protocols sent to the Centers in question. Finally each Centre initiating a scientific project (not a single Center project) undertakes to provide the Scientific Committee with an annual scientific report of its project.

**Article – 6.3. General conditions of extraction and use of samples from the Centers’ local collection**

A Centre that wishes to extract or to use the samples of the biocollection from one or more other Centers, must make a written request (email or letter) to the Coordinator. This request also includes a description of the scientific project. The said extraction or use can only be performed after written agreement (email or letter) from the Centre or Centers from which the data originate and that of the Scientific Committee.

Each Centre initiating a scientific project based on the use of samples from the biocollection undertakes to find the necessary funding to complete its project and to implement all the regulatory procedures necessary to ensure the compliance of its project with the legislation/regulations in force, and thus request all opinions, declarations, authorizations and decisions required. Studies made jointly between several Centers must be subject to written methodological protocols sent to the Centers in question. Finally each Centre initiating a scientific project (not a single Center project) undertakes to provide the Scientific Committee with an annual scientific report of its project.

Initials CHU Nantes Page 6 / 19 initials Parties Created by CJ CHUN

**Article – 7. Studies, Intellectual property and Publications**

For all projects or work carried out within the context of this Agreement, a special contract may be drawn up and concluded between the participating Centers, and, where appropriate, with third parties laying down in particular the conditions of collaboration or participation by the Centers, the budget, the

traceability of the data, and the apportionment of intellectual property rights. These contracts must refer to this Agreement.

Any publication or presentation (except for the single Centre projects) must mention the UH of Nantes's financial support (AOI 2015) for carrying out the project and then all the financial support of the Network (institutional or private), if any.

Any presentation or publication of a project undertaken with the assistance of a biological resource center (BRC) of one of the other Parties must include thanks to the aforementioned BRC or, where applicable, details of the BRC's assistance in the publication.

The Head of the BRC in question must be notified of every communication/publication produced, as well as every patent registered in cooperation with the BRC, in order to monitor its activity.

The stipulations of this article and those of Article 9 will not impede the obligation incumbent on each of the Parties to produce an activity report for the establishment to which they belong, as well as the defense of researchers' theses or reports/dissertations by students whose scientific activities are related to the purpose of this Consortium. These disclosures may be organized in closed sessions whenever necessary.

**Article - 7.1. Studies and work conducted jointly between several Centers and followed by publications**

- - 1. – Publications:

The publications produced in connection with the extraction or use of the EKiTE data and/or of the biocollection of one or several Centers will mention in the list of authors the Centre initiating the study and all the Centers involved in the study (one author per Centre) as hereinafter: first, second, third, before last and last author position in the publication will be attributed regarding scientific involvement of each Centre, followed by the collaborating Centers in proportion to the number of patients included in the database in descending order.

The prior written agreement (email or letter) of all the participating Centers must be obtained before publication.

Any such publication must mention the EKiTE Network which is coordinated by the UH of Nantes.

Any such publication/communication must mention in the acknowledgments all the research staff involved in the project (data managers, statisticians, clinical research associates….)

Any such publication/communication must mention financial support (institutional or private).

- - 1. – Intellectual property:

The results obtained by the Centers prior to any project carried out within the context of the Network, remain their respective property.

The results, relating to a project carried out independently of the Network belong to the Centers which have obtained them. The other Centers receive no rights in relation to the corresponding intellectual property titles because of this agreement.

The results of a project conducted within the context of the Network, pursuant to Articles 6.2 (paragraph 2 & 3) and 6.3, belong jointly to the Centers which have contributed to obtaining them, in proportion to their respective intellectual contributions. The same rules will apply to the allocation of property rights over the results of the work.

Initials CHU Nantes Page 7 / 19 initials Parties Created by CJ CHUN

**Article - 7.2. Studies and work conducted by a single Centre and followed by publications**

The Centers can freely extract and/or use their own data for their own projects.

- 1. ·1: Publications:

Publications by each Centre will be made under the signature of the Centre initiating the project. Any publication must mention the EKiTE Network which is coordinated by the UH of Nantes.

- - 1. – Intellectual property:

The property rights for the results of the work will belong to the Centre initiating the project.

**Article - 7.3. Studies and work carried out by academic or private third parties.**

Projects may be carried out by the Centers in collaboration with private or academic third parties.

In order to allow academic or private third parties to anticipate the protection of their projects and avoid any conflicts relating to confidentiality, the Centre approached by the said third party to carry out a project including the data and/or samples from the EKiTE Network, must inform it of the fact that, given its structure in the form of a Network, the third party's project will be presented to all the Centers via the Scientific Committee. Each project by a third party must be subject to a written methodological protocol sent to the Coordinator.

Each project will be discussed by the Scientific Committee, which will authorize or refuse the implementation of the said protocol, and will decide on the provisions for collaboration of the Network and of the Centers concerned for each project.

In all cases, when the work proposed by academic or private third parties requires the extraction or the use of all or part of the EKiTE database and/or the use of all or a part of the samples of the Centers, all the Centers will be informed in advance. Each Centre from which the data and/or samples used within the context of the said project originate, will, prior to it being carried out, give its written agreement (email or letter).

In the event of competition between a project proposed by a Centre of the Network and a project proposed on the initiative of a third party, the Scientific Committee will make a decision according to the scientific quality of the projects presented to it.

In all cases, for each project undertaken on the initiative of an academic or private third party with the Network, a special contract must be signed between the Centers involved and the third party. In particular this contract shall specify the conditions of collaboration or participation, the budget, the traceability of data and biological samples, and the apportionment of intellectual property rights and publications.

Any publication must mention the EKiTE Network which is coordinated by the UH of Nantes.

**Article – 8. Financial and administrative stipulations**

The EKiTE Network has a budget, the details of which are given in Annex 1.

**Article 8.1. Staff resources**

The Network requires staff resources.

The first three years, the UH of Nantes will fully support the database development and data management staff resources (CRA, data managers, coordinator…) based on the starting budget outlined in Annex 1. The staff resources within the context of the Network, will be subject to an assessment by the Scientific Committee which will decide, depending on the activity of each Centre in the cohort and the shared virtual/centralized biocollection, on the need to continue or pool between several Centers, the staff resources. The Scientific Committee will make a financial estimate and will assess the possible funding arrangements.

Initials CHU Nantes Page 8 / 19 initials Parties Created by CJ CHUN

In general, the assessment of staff resources is made and is decided by the Scientific Committee each year. Then, based on a proposal by the Scientific Committee, each Centre decides on the hiring or assigning arrangements, and the management of the staff required to participate in the Network, particularly with respect to the funding it has available. Each Centre is responsible for funding projects and related work that it wishes to carry out or in which it wishes to participate.

The UH of Nantes, because of its capacity of coordinator, shall bear any financial burden relating to staff whose intervention is necessary for the operation of the Network in the Centers, in addition to its own practitioners and agents and the costs outlined in the starting budget in Annex 1.

**Article 8.2. Material resources**

The Network requires material resources. Each Centre makes it its own business to fund and manage the material resources necessary for its collaboration in the EKiTE Network and for the performance of this agreement.

The operation of the EKiTE Network also involves the establishment of technical infrastructure and the provision by all the Centers of computer tools as well as storage equipment for the shared virtual biocollection. The UH of Nantes has therefore undertaken to:

- - - - Subscribe to a dedicated server for the EKiTE portal, including the Windows 2008 Server license, the Oracle license, the backup system and software, installation and configuration;
      - Subscribe to a hosting contract for health data in a Data Centre designed to meet the highest security requirements;
      - Subscribe to a corrective and scalable maintenance contract for the computer system for its own Centre.

The Nantes University Hospital manages the material resources required for the operation of the Network, limited to the resources listed above, with respect to each grant obtained for EKiTE. In general, the assessment of material resources is made and is decided by the Scientific Committee each year. Then, on a proposal by the Scientific Committee, the UH of Nantes decides on the arrangements for the acquisition, maintenance and/or management of materials required for the continuation of the Network corresponding to those already identified above, with respect to the funding available to it, obtained within the context of the Network.

In any event, the UH of Nantes will not be required to continue with the acquisition, maintenance and/or management of materials for which it does not have specific funding sources.

If there is a lack of funding, the Parties will consult each other to decide on the arrangements for continuing the Network and this Agreement. The lack of funding within the context of the Network may result in the early termination of this Agreement.

**Article – 9. Confidentiality**

As a reminder, in order to allow academic or private third parties to anticipate the protection of their projects and avoid any conflicts relating to confidentiality, the Centre approached by the aforementioned third party to carry out a study including the data and/or samples from the EKiTE Network, must inform it of the fact that, given the structure of the Network, the third party's project will be presented to all the Centers via the Scientific Committee.

Also, each Centre will undertake all necessary measures to protect information of a confidential nature relating to the other Centers, the disclosure of which may be made necessary for the proper performance of this Agreement. Under the terms of this Agreement, the Centers agree that this information may be written, verbal, digital or graphic, whatever the medium and the mode of transmission, and is considered as not publicly and legitimately available.

Initials CHU Nantes Page 9 / 19 initials Parties Created by CJ CHUN

This information is referred to hereinafter as "Confidential Information". It includes all information, knowledge, know-how or data of an intellectual, technical, scientific, commercial, financial or industrial nature, as well as any information relating to the organization of one of the Parties, its policy,

administrative and financial management, as well as all internal, accounting, social or legal information contained either in a written or electronic document or transmitted either verbally or visually, by examining items or equipment. "Confidential Information" also includes the various reports, as well as all information relating to the studies and work and the unpublished results of the latter.

Each Centre acknowledges and agrees that it may, limited to situations where the performance of this Agreement so requires, have access to the Confidential Information belonging to the other Centers and undertakes (i) not to disclose, discuss, supply, pass on, copy, make available or communicate, in any way, directly or indirectly, all or some of this Confidential Information of this Centre or Centers to a third party, without obtaining the prior agreement of this Party and (ii) not to use the Confidential Information belonging to this Party for a purpose other than that of exercising its rights and fulfilling its obligations resulting from this Agreement.

Notwithstanding these stipulations, either Party may pass on Confidential Information where it is able to provide evidence:

- that it was publicly available prior to or after it was passed on, without any wrongdoing on its part;
- that it has been received from a third party legitimately;
- that it was already in its possession prior to the conclusion of the Agreement, without any wrongdoing on its part;
- that it has been developed independently and in good faith by members of its staff who did not have access to this Confidential Information.

Nevertheless, the Party finding itself in possession of Confidential Information due to the aforementioned circumstances, undertakes to inform the Party in question of this, and in response to a request from the latter, to suspend communication/publication of any such Confidential Information in accordance with its instructions.

Moreover, each Party undertakes to:

- take all the necessary and reasonable measures and precautions to prevent Confidential Information belonging to the other Party and exchanged between them being disclosed to a third party;
- to pass on Confidential Information provided by the other Party to members of its staff or to its sub-contractors only where the latter need it in order to perform their duties arising from this Agreement, on condition that these members of staff are governed by professional secrecy, and that these sub-contractors are subject to a confidentiality agreement, covering the obligations stipulated in this Agreement and guaranteeing the secrecy and confidentiality of the Parties' Confidential Information;
- take all reasonable measures to prevent the staff from using Confidential Information belonging to the other Party for purposes other than the purpose of this Agreement;
- immediately notify the other Party of the occurrence of any event or the observation of any kind of activity such as to endanger the confidentiality of the Confidential Information, in order that the Parties are able to agree on precautions or other preventive measures to be implemented in order to preserve the secrecy of the Confidential Information.

The confidentiality undertaking forming the subject of this article must be mutually adhered to by the Centers for the entire term of this Agreement and for a period of *five (5)* years from the termination of this Agreement, with the exception of personal data, which is kept confidential for an unlimited period. On expiration of this Agreement, each Centre undertakes to return to the other Centers, all Confidential Information that it may have received or exchanged, in response to a request from the Centre in question, and in all cases, within thirty *(30)* days following the expiration, the termination or the exclusion of a Centre from the Consortium. The Centers undertake to provide each other with notification of destruction. Nevertheless, each Centre may retain one copy of the Confidential Information needed to fulfil its statutory monitoring and archiving obligations. In this case, the aforementioned Confidential Information is stored under conditions guaranteeing its strict confidentiality.

Initials CHU Nantes Page 10 / 19 initials Parties Created by CJ CHUN

As a reminder, CHU de Nantes may be allowed access to certain Confidential Information pursuant to its financial support, in particular reports.

In all cases, the Centers undertake to respect the principles relating to the protection of persons, in particular 95/46-CE European Directive and its implementing texts.

**Article – 10. Termination, exclusion**

Each Party may terminate this Agreement insofar as it binds it respectively and individually to the other Parties, subject to the observance of a notice period of six (*6*) months from the date of receipt of the notification sent to the other Parties. The data collected from the Party ending its collaboration will be extracted from the EKiTE database and communicated to the outgoing Centre according to its express instructions. They will also be retained in the EKiTE database. With regard to this Centre's samples, excluding cases where these have already been used, they will be stored in the Centre who received it or returned to the Centre in question, according to its instruction.

A Centre may be excluded from EKiTE and therefore this Consortium, because of the non-performance of the obligations stipulated in this Agreement. This exclusion must be ordered by a majority of the votes of the Scientific Committee. The decision ordering the exclusion of the Centre must be contained in a notification giving reasons and signed by all the other Centers sent to the latter. The data collected by the excluded Centre will be extracted from the EKiTE database and communicated to the excluded Centre according to its express instructions. They will also be retained in the EKiTE database. With regard to this Centre's samples, excluding cases where these have already been used, they will be stored in the Centre who received it or returned to the Centre in question, according to its instruction.

This Agreement may be terminated automatically should the Project be stopped for scientific or therapeutic reasons, in the event of a request issued by the competent health authorities or for any other reason that may be qualified as constituting force majeure.

In the event of termination or exclusion, the relevant Centers will lose their access to the EKiTE database. The amending decisions of this Agreement will have to be taken unanimously and be included in an express addendum.

**Article – 11. Term**

This Agreement is concluded for a period of 15 years as of the date of signature by all Parties. It may be renewed at the end of this period by an addendum concluded between the Parties.

Notwithstanding the expiration of this agreement, its early termination in the cases provided for in Article 10 "Termination - exclusion", the stipulations laid down in Article 9 shall remain in force for the period specified in the said article.

**Article – 12. Transfer**

This agreement is **non-transferable**.

**Article – 13. Entirety and limits of the Agreement**

This Agreement, with its annexes, expresses the entirety of the Centers’ obligations. None of the stipulations of this Agreement may be modified, altered, added to, amended or rearranged, either in whole or part, except by means of a document signed by the representatives duly authorized by the Parties. In the event of any inconsistencies between the terms of this Agreement and the terms of the annexes, the terms of this Agreement shall prevail.

Initials CHU Nantes Page 11 / 19 initials Parties Created by CJ CHUN

**Article – 14. Invalidity of a clause**

Should one or more clauses of this Agreement be regarded as invalid or declared as such pursuant to a treaty, law or regulation, or even as the result of a final decision by a competent court, the other clauses will retain all their force and scope. In this case, the Centers will immediately make the necessary changes in line, as far as possible, with the intentions existing at the time that this Agreement was signed.

**Article – 15. Disputes**

In the event of a difficulty about the interpretation or performance of this Agreement, the Centers will endeavour to resolve their dispute by mutual agreement. In the event of disagreement lasting for more than forty-five (45) days from the first notification sent by the complainant Centre to the Defaulting Party, the courts of the defendant will be referred to.

**Article – 16. Notification.**

Any notification regarding this Agreement which has to be sent by one Party to another Party will be sent by registered letter with acknowledgement of accept. The date of receipt is authoritative. As a reminder, notifications must be sent to the following addresses, namely:

For Nantes University Hospital:

- - Medical Affairs, Strategy and Research Department, for the attention of the Director of Medical Affairs and Research, 5, allée de l’Ile Gloriette, 44093 Nantes Cedex
  - Copy to Ms. Magali Giral, ITUN, 5, allée de l’Ile Gloriette, 44093 Nantes Cedex, France
    - - For Oslo University Hospital:
        - Chief Executive Officer, for the attention of Mr BJØRN ERIKSTEI
        - Copy to Mr. Anders Åsberg, Department of Transplant Medicine, Clinic for Surgery, Inflammation and Transplantation, Oslo University Hospital – Rikshospitale, P.O. Box. 4950, Nydalen, 0424, Oslo, Norway
      - For Leuven University Hospital:
        - Chief Executive Officer, for the attention of Mr MARC DECRAMER
        - Copy to Mr. Maarten Naesens Nephrology department
      - For Bellvitge University Hospital:
        - Chief Executive Officer, for the attention of Mr ANTONI LLUÍS ANDREU PÉRIZ
        - Copy to Mr. Oriol Bestard Nephrology department

Initials CHU Nantes Page 12 / 19 initials Parties Created by CJ CHUN

**For the UH of Nantes**

Mr Philippe SUDREAU

# Chief Executive Officer

And per pro.

Ms Anne-Claire de Reboul

# Director of Medical Affairs and Research

In the presence of

# Pr Magali Giral Coordinator

**Nantes, ……./……………../…………….**

Initials CHU Nantes Page 13 / 19 initials Parties Created by CJ CHUN

**For the UH of Leuven**

Mr Marc DECRAMER

# Chief Executive Officer

In the presence of

# Dr Maarten Naesens Scientific Manager

**Leuven, ……./……………../…………….**

Initials CHU Nantes Page 14 / 19 initials Parties Created by CJ CHUN

**For the UH of Oslo**

Mr Bjørn ERIKSTEIN

# Chief Executive Officer

In the presence of

# Pr Anders Åsberg Scientific manager

**Oslo, ……./……………../…………….**

Initials CHU Nantes Page 15 / 19 initials Parties Created by CJ CHUN

**For UH of Barcelona**

Mr [Antoni Lluís Andreu PÉRIZ](http://www.bellvitgehospital.cat/staff/view.php?ID=1682)

# Chief Executive Officer

In the presence of

# Dr Oriol Bestard Scientific Manager

**Barcelona, ……./……………../…………….**

Initials CHU Nantes Page 16 / 19 initials Parties Created by CJ CHUN

| **Annex 1– EKiTE fixed starting budget** | | | | | | | |
| --- | --- | --- | --- | --- | --- | --- | --- |
|  | **Budget** | | | | | |  |
|  | **University Hospital of Nantes** | **Coordinator: Pr Magali Giral** | | **Projet n° : RC15_0419** | | |  |
|  | **TYPE OF EXPENSE** | **DETAILS** | **2016** | **2017** | **2018** | **Total In €** |  |
|  | **Promotion fees** | Creation of dossier | 900 | 0 | 0 | 900 |  |
|  | **STAFF** | |  |  |  |  |  |
|  | Medical staff | Pharmacovigilance | 0 | 0 | 0 | 0 |  |
|  |  | Medical expertise | 0 | 0 | 0 | 0 |  |
|  | Non-medical staff | Monitoring | 0 | 0 | 0 | 0 |  |
|  |  | Administration | 300 | 900 | 900 | 2100 |  |
|  |  | Technician | 0 | 0 | 0 | 0 |  |
|  |  | Network manager | 26000 | 10400 | 10400 | 46800 |  |
|  |  | Software development | 39480 | 0 | 0 | 39480 |  |
|  |  | Biostatistician / data management | 0 | 43140 | 0 | 43140 |  |
|  | *Sub-total (1)* |  | 66680 | 54440 | 11300 | 132420 |  |
|  | **MEDICAL EXPENSES** | |  |  |  |  |  |
|  | Pharmacy |  | 0 | 0 | 0 | 0 |  |
|  | Small medical material |  | 0 | 0 | 0 | 0 |  |
|  | Laboratory supplies |  | 0 | 0 | 0 | 0 |  |
|  | Laboratory reagents |  | 0 | 0 | 0 | 0 |  |
|  | Imaging |  | 0 | 0 | 0 | 0 |  |
|  | Sub-contracting of a medical nature |  | 0 | 0 | 0 | 0 |  |
|  | Maintenance of a medical nature |  | 0 | 0 | 0 | 0 |  |
|  | Serum Bank |  | 0 | 0 | 0 | 0 |  |
|  | *Sub-total (2)* |  | 0 | 0 | 0 | 0 |  |
|  | **GENERAL EXPENSES** | |  |  |  |  |  |
|  | Publication fees |  | 0 | 0 | 2000 | 2000 |  |
|  | General fees (telephone/fax/supplies) |  | 200 | 0 | 0 | 200 |  |
|  | Fixed administrative fees |  | 0 | 0 | 0 | 0 |  |
|  | Archiving |  | 0 | 0 | 0 | 0 |  |
|  | Small hotel |  | 0 | 0 | 0 | 0 |  |
|  | Stationary |  | 0 | 0 | 0 | 0 |  |
|  | Small material |  | 0 | 0 | 0 | 0 |  |
|  | Desk and informatics supplies |  | 0 | 0 | 0 | 0 |  |
|  | Lease of IT equipment |  | 0 | 0 | 0 | 0 |  |
|  | Equipment rental |  | 0 | 0 | 0 | 0 |  |
|  | Maintenance and repair |  | 0 | 0 | 0 | 0 |  |
|  | Insurances |  | 0 | 0 | 0 | 0 |  |
|  | Documentation |  | 0 | 0 | 0 | 0 |  |
|  | External services |  | 0 | 0 | 0 | 0 |  |
|  | Transport of patients |  | 0 | 0 | 0 | 0 |  |
|  | Staff outside of the establishment |  | 0 | 0 | 0 | 0 |  |
|  | Travelling |  | 5000 | 5000 | 5000 | 15000 |  |
|  | *Sub-total (3)* |  | 5200 | 5000 | 7000 | 17200 |  |
|  | **FINANCIAL EXPENSES** | |  |  |  |  |  |
|  | Financial fees (medical mat.) |  | 0 | 0 | 0 | 0 |  |
|  | Extraordinary charges |  | 0 | 0 | 0 | 0 |  |
|  | Depreciation |  | 0 | 0 | 0 | 0 |  |
|  | *Sub-total (4)* |  | 0 | 0 | 0 | 0 |  |
|  | **TOTAL (1)+(2)+(3)+(4)** |  | 71880 | 59440 | 18300 | 149620 |  |

**Annex 2 – Listing of the database items**

# Variables related to the recipient of the surgery

- - - - - Year of transplantation
        - Recipient gender
        - Recipient age
        - Recipient height
        - Recipient weight
        - Rank of current kidney transplantation
        - Duration under renal replacement therapy
        - Duration on waiting list before the transplantation
        - Type of dialysis just before the transplantation
        - Primary renal disease
        - Vascular history
        - Cardiac history
        - Cancer history
        - Diabetes history
        - Hepatitis history
        - Recipient CMV serology
        - Recipient EBV serology
        - Anti-class I Immunization
        - Anti-class II Immunization
        - Recipient HIV serology
        - Recipient blood group
        - Induction therapy

# Variables related to the donor at the surgery (no follow-up donors for living donations)

- - - - - Age at the graft retrieval
        - Donor gender
        - Type of donor
        - Type of deceased donation
        - Donor cause of death
        - Last donor serum creatinine
        - Donor CMV serology
        - Donor EBV serology
        - Donor blood group

# Variables related to the transplantation at the surgery

- - - - - Cold ischemia time
        - Number of HLA-A mismatches
        - Number of HLA-B mismatches
        - Number of HLA-DR mismatches

# Variables collected during the post-transplantation period

- Recipient weight
- Maintenance immunosuppressive drug (7 subtypes)
- Recipient serum creatinine
- Recipient daily proteinuria
- Delayed graft function
- Time-to-death
- Time-to-graft failure
- Time-to-first acute rejection episode (ARE)
- Maximum follow-up time

# From the above variables, missing data is not allowed for the following variables:

**Variables related to the recipient of the surgery**

- Year of transplantation
- Recipient gender
- Recipient age
- Rank of current kidney transplantation
- Time-to-death

# Data collected during the post-transplantation period

- Time-to-graft failure
- Time-to-first acute rejection episode (ARE)
